# Assessment of hepatocellular carcinoma risk based on peg-interferon plus ribavirin treatment experience in this new era of highly effective oral antiviral drugs: Erratum

**DOI:** 10.1097/MD.0000000000006193

**Published:** 2017-02-17

In the article “Assessment of hepatocellular carcinoma risk based on peg-interferon plus ribavirin treatment experience in this new era of highly effective oral antiviral drugs”,^[1]^ which appeared in Volume 96, Issue 1 of *Medicine*, an acknowledgment read “This study was supported by an Inha University Hospital grant”. The statement should have read “This study was supported by an Inha University Research grant”.
